# COVID-19 Disease in Infants Less Than 90 Days: Case Series

**DOI:** 10.3389/fped.2021.674899

**Published:** 2021-07-12

**Authors:** Lana A. Shaiba, Khalid Altirkawi, Adnan Hadid, Sara Alsubaie, Omar Alharbi, Hamad Alkhalaf, Musaed Alharbi, Nourah Alruqaie, Omar Alzomor, Fahad Almughaileth, Nasser Alyousef, Prakesh S. Shah

**Affiliations:** ^1^Pediatric Department, College of Medicine, King Saud University, Riyadh, Saudi Arabia; ^2^Neonatal Intensive Care Unit, King Saud University Medical City, Riyadh, Saudi Arabia; ^3^Pediatric Infectious Diseases Unit, King Saud University Medical City, Riyadh, Saudi Arabia; ^4^Department of Pediatrics, King Saud Bin Abdulaziz University for Health Sciences, Riyadh, Saudi Arabia; ^5^King Abdullah International Medical Research Center, King Abdulaziz Medical City, Riyadh, Saudi Arabia; ^6^Department of Pediatrics, King Abdullah Specialist Children's Hospital, Riyadh, Saudi Arabia; ^7^Pediatric Infectious Diseases Department, Children Hospital, King Saud Medical City, Riyadh, Saudi Arabia; ^8^Department of Pediatrics, Mount Sinai Hospital, Toronto, ON, Canada; ^9^Department of Pediatrics, Toronto University, Toronto, ON, Canada; ^10^Maternal-Infant Care Research Centre, Mount Sinai Hospital, Toronto, ON, Canada

**Keywords:** COVID-19, infants, MIS-C, SARS-CoV-2, 90 days

## Abstract

The objective of this study is to describe the clinical presentations, radiological and laboratory findings, and outcomes of COVID-19 disease in infants ≤ 90 days of age at presentation. We conducted a retrospective study of infants in this age group who were found to be SARS-CoV-2 positive. Asymptomatic infants who were identified through routine testing following delivery to COVID-19-positive mothers were excluded. We classified infants according to their presentation: asymptomatic, mildly symptomatic, moderately symptomatic, and severely/critically symptomatic. A total of 36 infants were included. Of them, two were asymptomatic and four had severe/critical presentation. Of the severely symptomatic infants, two were considered as multisystem inflammatory syndrome in children (MIS-C) and there was one death. One infant in the severe symptomatic group presented with cardiac failure, with the possibility of congenital infection. Another infant presented with cardiogenic shock. None of these infants received antiviral medication. The study found that infants ≤ 90 days can present with a severe form of COVID-19 disease. Multisystem inflammatory syndrome in children, although rarely reported in infants, is a possible complication of COVID-19 disease and can be associated with significant morbidity and mortality.

## Introduction

COVID-19 disease can assume various clinical presentations. Patients may be asymptomatic or have mild to severe symptoms, and in some cases, the disease can even be fatal (1). Although children can be affected by COVID-19, they are less likely to be symptomatic or to develop severe symptoms than adults. Furthermore, the disease seems to be less common in infants younger than 1 year of age; its incidence in this age group is estimated to be 12–18% (2). It is still unclear why children are less likely to be affected by this novel virus. However, it has been linked to the low expression of angiotensin-converting enzyme 2 (ACE2) receptors, which seem to be the main entry point of the virus into the host system (3).

Broadly, most of the symptoms of COVID-19 disease in children are respiratory and gastrointestinal (4), although more nuanced accounts of the clinical and laboratory features of COVID-19 in children, especially young infants, are limited (5). Indeed, only a few studies have described the clinical disease in infants younger than 90 days of age. Of note, a population-based study in the United Kingdom described a more severe form of the illness in neonates affected with COVID-19 (6). Seemingly, most of the confirmed cases in children were from familial clusters or had close contact with COVID-19 patients (7).

With the first wave of this COVID-19 pandemic, the Kingdom of Saudi Arabia was moderately affected, with 364,754 cases reported until January 17, 2021. Among these patients, a total of 72,999 (20%) were children, of whom 2,128 (2.9%) were younger than 1 year of age. As is the case elsewhere, there is a scarcity of information describing the local burden of COVID-19 disease in children, especially in infants and neonates (8, 9). In this study, we aim to describe the clinical characteristics, course, and outcomes of SARS-COV-2 infection in a local population of infants ≤ 90 days of age.

## Methods and Settings

### Study Design, Participants, and Settings

We conducted a retrospective analysis of data collected on infants admitted to three tertiary hospitals in Riyadh, Saudi Arabia, from March 1, 2020 to August 31, 2020 who were ≤ 90 days of age with a positive nasopharyngeal swab (NP) for SARS-CoV-2. The three hospitals were King Saud University Medical City, a university hospital; King Abdullah Specialist Children's Hospital, a National Guard hospital; and King Saud Medical City, a tertiary-care hospital governed by the Ministry of Health.

### Ethical Approval

The Institutional Review Boards of the three hospitals approved the study and exempted it from requiring consent. The King Saud University Medical City registration number is 20/0689/IRB (E-20-5262), the King Abdullah International Medical Research Center reference number is RC20/454/R, and the King Saud Medical City registration number is H1R1-25-Aug20-04.

The investigators performed separate electronic record reviews from the databases of each of the contributing hospitals. Later, the extracted data were merged into a single set for final analysis.

### Case Identification

Infants were included if they were ≤ 90 days of age and had at least one positive NP for SARS-CoV-2 virus with polymerase chain reaction (PCR) testing. Infants were subjected to NP swabs for detecting the viral genome of SARS-CoV-2 for (1) presenting with clinical symptoms suggestive of COVID-19 disease, (2) history of contact with a positive SARS-CoV-2 household identified upon a health encounter due to unrelated illnesses, or (3) showing signs compatible with COVID-19 disease after being born to a mother who had active COVID-19 disease during the intrapartum period. We excluded infants with positive SARS-CoV-2 virus PCR test if they were asymptomatic and their testing was performed due to maternal SARS-CoV-2 positive status at delivery.

A confirmed case of COVID-19 was defined as a positive result for both SARS-CoV-2 E and S genes using the RealStar® SARS-CoV-2 real-time reverse transcriptase PCR (RT-PCR) kit (Altona®-Diagnostics, Hamburg, Germany) and Rotor-gene Q system (Qiagen®, Santa Clarita, CA, USA). We confirmed that a sample was a positive case with a cycle threshold (*Ct*) value ≤ 29 for both SARS-CoV-2 E and S genes. A sample with a single gene detection or a *Ct*-value ≥29 was confirmed by repeating the test on the Xpert® Xpress SARS-CoV-2 kit and the GeneXpert XVI system (Cepheid®, Sunnyvale, CA, USA), which detect SARS-CoV-2 E and N genes. All samples were collected in a negative-pressure room in accordance with hospital policies and guidelines.

### Definition of the Disease

Initially, we divided patients into two groups: asymptomatic and symptomatic. Asymptomatic patients were infants without symptoms suggesting a viral infection but who were found to have a positive SARS-CoV-2 PCR upon presentation and were tested due to history of contact with COVID-19-confirmed cases. Symptomatic infants were those presenting with symptoms suggesting a viral illness and with a positive SARS-CoV-2 virus PCR. Using a clinical definition for disease severity adopted from Dong et al. (2), we further divided the disease of symptomatic patients into:

Mild disease: Presented with fever, runny nose, or dry cough with no tachypnea or respiratory distress, or presented with gastrointestinal symptoms of vomiting and diarrhea without signs of dehydration.

- Moderate disease: Presented with clinical and radiological findings of pneumonia without hypoxemia.
- Severe/critical disease: Presented with evidence of hypoxemia and low arterial oxygen saturation (SpO_2_ <92%) and tachypnea, respiratory distress, respiratory failure, shock, multisystem failure, coagulopathy, acute kidney injury, myocardial injury, or encephalopathy.

### Statistical Analysis

For descriptive statistics, we classified patients into four groups based on severity of illness at time of presentation (asymptomatic, mild, moderate, and severe/critical). Also, we compared variables of demographics, presentation, laboratory, imaging results, and management issues based on patients' age categories (neonatal vs. post-neonatal). χ^2^-test and *t*-test for independent samples were used, as appropriate, to assess differences between groups. *P*-values of < 0.05 were considered significant. We reported results of continuous variables as mean (or median) and range, whereas categorical variables were reported as percentages. Statistical analysis was performed using IBM SPSS statistics version 22.0 software (IBM Inc., Armonk, NY, USA).

## Results

**Figure 1:**
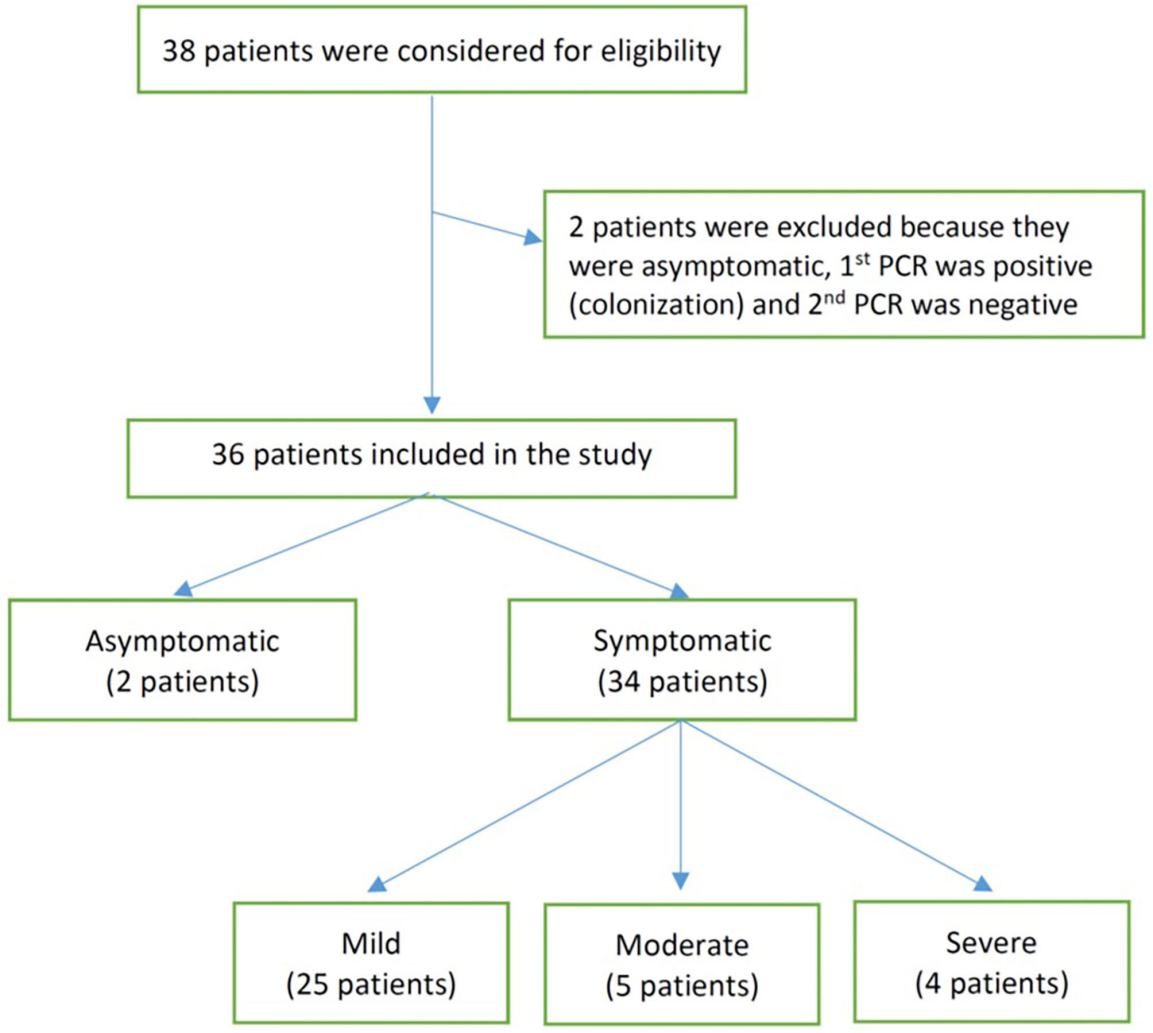
Flow chart shows the numbers of individuals at each stage of study.

We identified 38 infants born between March 1, 2020 and August 31, 2020 from three major birthing centers in Riyadh, Saudi Arabia. Of those, 36 met our inclusion criteria and were included for analysis (Figure 1). The included infants had ages ranging between 1 and 90 days at the time of testing for SARS-CoV-2. Of the infants included, 2 were asymptomatic (tested due to exposure to a household member positive for SARS-CoV-2), 25 had mild disease, 5 had moderate disease, and 4 had severe/critical disease (see Table 1). At presentation, the COVID-19 PCR status of mothers was positive for all the asymptomatic infants, 48% of the mild group (12 COVID-19-positive mothers), 40% of the moderate group (2), and 25% of the severe/critical group (1). Breastfeeding was initiated before admission in most of the infants, including 2 (100%) of the asymptomatic group, 14 (56%) of the mild group, 3 (60%) of the moderate group, and 2 (50%) of the severely ill group. The median duration of symptoms before presentation was 1 day in the mild and moderate groups and 3 days in the severe group. All the asymptomatic, moderately, and severely ill infants were admitted, while only 15 (60%) of the mildly ill infants were admitted. The length of hospital stay was, as expected, longer in infants who were categorized as severe/critical (see Table 1 for more details).

**Table 1:** Clinical and laboratory characteristics of COVID-19 in early infancy.

	**Asymptomatic**	**Mild**	**Moderate**	**Severe/Critical**
*N*	2	25	5	4
Age at presentation, days; mean (range)	38 (15–61)	50 (1–90)	18 (1–90)	21.5 (1–90)
Male, *n* (%)	1 (50)	12 (48)	5 (100)	2 (50)
Spontaneous vaginal delivery, *n* (%)	2 (100)	16 (64)	5 (100)	4 (100)
Mother age, years; mean (range)	25 (22–29)	31 (20–43)	27.5 (22–41)	34
Gestational age, weeks; mean (range)	39 (39–41)	38 (23–40)	38 (38–39)	37 (37–37)
Birthweight, g; mean (range)	3,395 (2,530–3,600)	3,144 (2,530–3,800)	2,815 (2,800–3,670)	3,550 (2,500–4,600)
Mother's COVID-19 status at presentation, positive, *n* (%)	2	12	2	1
Breastfeeding, *n*	2	14	3	2
Fever, *n* (%)	NA	18	4	3
Respiratory signs, *n*	NA	15	5	3
Gastrointestinal signs, *n*	NA	7	2	1
Number hospitalized	2	15	5	4
White blood cells; mean (range)	14 (10.9–17.9)	7.3 (2.8–12.6)	10.5 (6.9–12.5)	10.15 (6–29.4)
Platelet counts; mean (range)	259 (177–341)	327 (144–614)	380 (263–552)	231 (43–360)
Platelet <150	0	1	0	0
Platelet <100	0	0	0	1
Abnormal CXR	NA	14 (56)	5 (100)	4 (100)
Resource utilization and outcome				
O2 therapy, days; mean (range)	0	0	0	15 (14–75)
Length of stay in intensive care unit, days; mean (range)	0	0	0	12.5 (3–16)
Hospital length of stay, days; mean (range)	6 (5–8)	4 (2–10)	4 (2–7)	70 (15–75)
Mortality	0	0	0	1 (25%)

Out of the 36 infants in this cohort, four presented in critical or severe condition (Table 2). They were hospitalized for a median [range] of 70 [15–75] days, of which 12.5 [3–16] days were in the intensive care unit (ICU). Two of these were considered as multisystem inflammatory syndrome in children (MIS-C), based on the definition criteria of the World Health Organization, even if another infection could not be strictly excluded (10), The first patient presented with fever and gastrointestinal symptoms, and, at a later stage, hypothermia. Laboratory findings were significant for elevated cardiac enzymes (elevated troponin and Brain natriuretic peptide), impaired renal function (elevated urea and creatinine), and high inflammatory markers (elevated C-reactive protein, elevated ferritin, elevated D-dimer). Cultures obtained from blood, urine and tracheal aspirate were all reported with no growth. Given the critical condition of the patient a lumbar puncture was differed. The patient died after 2 weeks of hospitalization. The second patient had a week of fever and respiratory symptoms prior to presentation. He presented to the emergency department with shock signs, increased inflammatory markers (elevated C-reactive protein and ferritin), and cardiac enzymes (elevated troponin and BNP). Blood and urine culture were negative, so was the result of the respiratory viral panel. The respiratory aspirate culture has grown *Stenotrophomonas maltophilia*. A lumbar puncture was also differed in this patient. Both patients were managed with corticosteroids, intravenous immunoglobulin (IVIG), and human interleukin-1 receptor antagonist (anakinra), an immunomodulator agent.

**Table 2:** Characteristics and outcomes of severe/critical cases.

	**Patient #1**	**Patient #2**	**Patient #3**	**Patient #4**
Age (Days)	30	90	1	13
Sex	Female	Male	Male	Female
Length of hospital stay	15 days (all in ICU)	75 days (all in ICU)	75 days (18 days in ICU)	70 days (47 in ICU)
Outcome	Death	Survive and discharge home	Survive and discharge home	Survive and discharge home
Lab				
RT-PCR for SARS-CoV-2	1st PCR +ve at presentation, 2nd PCR +ve (10 days later)	1st PCR +ve at presentation, 2nd PCR –ve (29 days later)	1st PCR +ve, 2nd PCR +ve (6 days late)	1st PCR +ve, 2nd PCR –ve (4 days later)
Septic workup	Blood and urine CX negative, CSF not done	Blood and urine CX negative, CSF not done	Blood culture was negative, urine culture was positive (*Klebsiella* pneumonia), CSF not done	Blood and urine culture negative, CSF not done
White blood cell count x109/L	11.3 (7.02–58.6)	29.4 (5–29.4)	5.99 (5.99–12.2)	9 (3.57–32.7)
Neutrophil count x109/L	2.71 (2.71–28.05)	12.94 (2.16–22.4)	0.6 (0.6–4.51)	5.24 (0.56–11)
Lymphocyte count x109/L	6.78 (1.12–16.41)	10.29 (0.59–10.29)	3.53 (3.37–9.18)	2.29 (1.6–4.56)
Platelet count x109/L	43 (29–427)	221 (29–121)	360 (201–575)	242 (130–639)
Creatinine	216 (max 249)	79 (max 79)	47 (max 81)	71 (max 72)
Blood urea nitrogen	23 (max 23)	9.4 (max 9.4)	1 (max 8.8)	6.3 (max 7)
Sodium	123 (min 123)	139 (min 133)	136 (min 135)	147 (min 132)
Potassium	6.4 (min 2.8)	5.3 (min 2.7)	5.1 (min 3.2)	5.4 (min 3.5)
Lactate	1.7 (max 8.2)	3.2	2.7 (max 2.7)	1.8 (max 7.74)
Alanine transaminase	14 (max 74)	1,070 (max 1,070)	NA (max 24)	54 (max 54)
Aspartate transaminase	73 (max 143)	1,178 (max 1,178)	NA (max 36)	152 (max 152)
Albumin	31 (min 27)	32 (min 17)	32 (min 30)	32 (min 25)
Lactate dehydrogenase	1,580 (max 2,068)	1,105 (max 1,105)	NA	NA
Creatine kinase-MB	16.4 (max 16.4)	40.6 (max 40.6)	NA (max NA)	NA (max 94)
Troponin	684 (max 2,410)	108 (max 1,294.2)	46.3 (max 46.3)	NA (max 15,724.18)
Brain natriuretic peptide	971 (max 1,127)	1,370 (max 2,241)	117.8 (max 390)	NA (max NA)
Partial thromboplastin time	55.5 (max 118)	38.1 (max 52.8)	20.5 (max 61.8)	53 (max 117)
International normalized ratio	1.04 (max 1.55)	1.49 (max 1.85)	1.12 (max 3.37)	1.16 (max 1.67)
D dimer	5.5 (max 5.93)	1.32 (max 1.85)	NA (max NA)	5.89 (max 5.89)
fibrinogen	2.25 (max 2.25)	0.71 (max 0.55)	NA (max NA)	1.3 (max 2)
C-reactive protein	11 (max 22)	56 (max 137)	2 (max 2)	3 (max 31)
Erythrocyte sedimentation rate	2 (max 2)	2 (max 2)	2 (max 2)	2 (max 84)
Procalcitonin	1.7 (max 1.7)	0.08 (?)	0.09 (max 0.09)	0.06 (max 0.06)
ferritin	2,316 (max 6,130)	813 (max 813)	NA (max NA)	NA (max NA)
Interleukin 6	9	9	NA	NA
Tumor necrosis factor alpha	19	26	NA	NA
Chest x-ray	Bilateral opacities, pulmonary edema, atelectasis	Unilateral opacities, peribronchial thickening, pleural effusion	Pulmonary edema	Atelectasis
Echocardiography	Normal coronaries, small pericardial effusion EF mode 80.5	Mild TR, dilated RA, and RV, suprasystemic pulmonary hypertension, EF mode 60	Normal left ventricular systolic function, dilated left ventricle, large per membranous VSD	TGA moderate VSD, moderate ASD II
Antibiotics	Total of 12 days	Total of 18 days	Total of 10 days	Total of 4 days
Respiratory support	CMV 12 days, HFO 2 days	CMV 38 days, HFO 20 days, Bipap/CPAP 5 days	Nasal canula 75 days	Bipap/CPAP 5 days
Duration of O_2_ support	14 days	72 days	75 days	15 days
Other medication	Hydralazine, amlodipine, anakinra, and heparin	Hydralazine, amlodipine, anakinra, sildenafil, aspirin, and enoxaparin	Captopril, furosemide	Captopril, propranolol, furosemide
IVIG	2 doses (1 g/kg/dose)	2 doses (1 g/kg/dose)	Not given	Not given
Steroid	Given	Given	Given	Not given
Antiviral	None	None	None	None

The other two cases in the severe/critical group had an underlying cardiac disease. The first patient was born via spontaneous vaginal delivery to a COVID-19-positive mother and then developed respiratory distress requiring oxygen support. At a later stage, he developed cardiac failure secondary to congenital heart disease, atrial septal defect (ASD) and ventricular septal defect (VSD) that was managed by furosemide and captopril. The fourth patient presented to the emergency department with fever and respiratory distress and was diagnosed with transposition of great arteries (TGA).

As Table 2 illustrates, an abnormal chest x-ray was noted in all infants with severe/critical disease, and none of them received antiviral therapy.

Comparing infants based on their age at time of presentation revealed that there were no significant differences in presentation, laboratory features, or resource utilization between the neonatal (*n* = 16) and post-neonatal patients (*n* = 20) except for fever, as more post-neonatal infants were febrile at the time of presentation (see Supplementary Table).

Among the infants who had chest x-ray studies obtained (*n* = 23), 10 had normal studies and 13 had radiological abnormalities, including peribronchial thickening (7), atelectasis (2), opacities (1), pulmonary edema (1), multiple findings of opacities, pulmonary edema, and atelectasis (1), and opacities, peribronchial thickening, and pleural effusion (1).

All the infants included in the study were discharged from the hospital except for the one infant who died.

## Discussion

In agreement with current literature (11, 12). which reported milder COVID-19 disease in children, and that severe disease is more likely to occur in children with comorbidities, such as cardiac and pulmonary illnesses (12–14), this multicenter case series suggests that the majority of infants who are ≤ 90 days old present with mild disease, although nearly 10% of the subjects had severe or critical presentation. Respiratory symptoms were more prevalent in this cohort than gastrointestinal symptoms. Infants in both age groups—neonatal and post-neonatal—had similar presentation and outcome profiles. Of interest, all infants who were categorized as severely symptomatic in this cohort presented with abnormal chest x-rays.

Most of the patients in this study were mildly symptomatic, with fever being the most common presenting feature, particularly in the post-neonatal age group (30–90 days), in a resemblance to older children (2, 11, 12). Respiratory signs were present in all patients with moderate illness and in the majority of those with severe/critical illness. Of note, our data suggest that infants ≤ 90 days old may have more severe disease than what has been reported in older children: four infants in this cohort (almost 11%) met the criteria for severe/critical disease, in contrast with what has been previously reported: 6% in children below 18 years (2, 14). The severe course and need for critical care or respiratory support in some of these patients might be explained by the presence of two possible cases of MIS-C among the post-neonatal age group and the presence of other conditions, such as cyanotic heart disease and bacterial coinfections. Although the deterioration we identified in two of the severe/critical patients could be ascribed to their underlying cardiac conditions, it is more likely to be related to COVID-19 diagnosis. Conceivably, the pre-existing heart anomalies may have contributed to their worsening mandating ICU admission. However, one cannot totally separate the intertwined effects of the two conditions (COVID-19 and cardiac illness) and how they contributed to the patients' outcomes. Furthermore, the onset of new symptoms after the identification of SARS-CoV-2 infection makes it more likely to be related to COVID-19 disease. Reports from the UK on neonatal COVID-19 infection also confirmed a higher rate of severe disease in 42% of cases, and that 36% of the babies in these studies received care in a neonatal unit or pediatric ICU (6). However, 24% of babies in their series were born preterm, which could have influenced the need for ICU care.

MIS-C is a well-recognized disease associated with COVID-19 infection in children; however, its spectrum and sequala are not fully understood. Although the majority of reported cases are in children older than 1 month of age, a few reports described this condition in neonates (15–17). These reports indicated how challenging the diagnostic and therapeutic approaches were, and most of the patients required extensive courses of an immunomodulator agent (anakinra), IVIG, and corticosteroids (15–17). In the two cases of MIS-C presented in this study although viral myocarditis could not be excluded, given the temporality of isolation of SARS-CoV-2 in the neonate, we proposed that this can be due to SARS-CoV-2 induced effect on the myocardium in addition to the effect on other organs raising the possibility of MIS-C in the neonate.

The 16.5-days median age of presentation of the neonatal age group suggests that the majority of these patients have acquired the disease postpartum, possibly from exposure to an infected mother or another family member. This is potentially important since neonatal SARS-CoV-2 infections seem to occur more commonly through a postnatal environmental exposure than transplacental acquisition (18). The role of breastfeeding in COVID-19 transmission remains disputed; in our cohort, the affected babies in the post-neonatal age group were more likely to be breastfed compared to those in the neonatal age group. Breast-feeding by an infected mother remains a contested issue. While earlier studies during the pandemic supported the safety of breast milk, the detection of SARS-CoV-2 RNA in breast milk samples, as reported by recent studies, calls for concern (19–21). Therefore, the role of breast-feeding as a mode of transmission of SARS-CoV-2 requires further exploration.

In a report by Panetta et al. on infants younger than 1 year, 14 were below 3 months of age and 13 were 3–12 months of age. The report showed that most infants below 3 months presented with mild symptoms (86%), and only 8 of them were hospitalized (5). Interestingly, most of these infants presented with gastrointestinal symptoms, in contrast with our study, where the most common presenting feature was fever, followed by respiratory symptoms. Their study reported that none of the infants required ICU admission, and only 10 were hospitalized. This was not the case in our study, where all infants presenting with moderate and severe symptoms (*n* = 9) were hospitalized, and 17 of the asymptomatic/mild symptomatic infants were hospitalized.

Many factors may contribute to the finding of decreased severity and incidence of COVID-19 disease in children. These factors include the decreased expression of ACE2 receptor, the primary target of SARS-CoV-2 virus; the differences in transmembrane protease serine 2 (TMPRSS2) between children and adults; and the larger number of other viruses residing in the lung and airway mucosa, which could limit the replication of SARS-CoV-2 by direct virus-to-virus competition (3). Also, children may have stronger innate immune responses and fewer comorbidities, such as smoking and obesity (22, 23). Finally, fetal hemoglobin is speculated to have a role in protecting against coronavirus infection in neonates (24).

Another hypothesis to explain the decreased severity of COVID-19 in infants, as opposed to the elderly, is alterations at neurotransmitter level, especially of substance P (SP) and of the trigeminal nucleus in the brainstem that controls its secretion (25). Substance P, a neuropeptide that acts centrally and peripherally, is produced also in the respiratory system, especially in the bronchopulmonary C fibers, where it is expected to exert lung-protective effects. Compared to adults, neonates and infants up to 1 year of age express higher levels of SP, which renders them better protected when exposed to the SARS-COV-2 viral infection. Also this altered release of SP, through the trigeminal nerve, seems to contribute to the common symptoms of COVID-19 infection, such as headache, sore throat and loss of smell and taste.

Despite a fairly good number of infants being included in this report, larger studies are warranted to corroborate our findings and to help generalize these results. Furthermore, the retrospective nature of the study is invariably associated with a potential for bias, complicated by the heterogeneity of the patient recruiting process. Nonetheless, all PCR tests were performed under national approval for testing issued by the Ministry of Health and with the appropriate use of positive and negative controls.

## Conclusion

Although most reported case series of infants presenting with COVID-19 indicate mild forms of disease, it is still possible for these infants to present with severe forms, leading to significant morbidities and even mortality. Further studies at national and international level with larger sample sizes are still needed to elucidate the extent of this disease and its severity in neonates and young infants.

## Data Availability Statement

The original contributions generated for the study are included in the article/Supplementary Materials, further inquiries can be directed to the corresponding author/s.

## Ethics Statement

The studies involving human participants were reviewed and approved by King Saud Univ Med City. Written informed consent to participate in this study was provided by the participants' legal guardian/next of kin. The animal study was reviewed and approved by King Saud Univ Med City. Written informed consent was obtained from the owners for the participation of their animals in this study. Written informed consent was obtained from the individual(s), and minor(s)' legal guardian/next of kin, for the publication of any potentially identifiable images or data included in this article.

## Author Contributions

LS, the principal investigator and corresponding author, conceptualized the study, and finalized the manuscript. KA carried out the statistical analysis of the study, reviewed the results, and critically reviewed the manuscript. AH designed the data collection, reviewed, and revised the manuscript. SA shared in data collection and writing the discussion part of the manuscript. OAlh, OAlz, NAly, HA, FA, and NAlr contributed in data collection. MA contributed to data collection and manuscript preparation. PS has helped in the conceptualization of the study and critical review of the manuscript. All authors approved the final manuscript as submitted and agree to be accountable for all aspects of the work.

## Conflict of Interest

The authors declare that the research was conducted in the absence of any commercial or financial relationships that could be construed as a potential conflict of interest.
