# Supplementary material for: COVID-19 Disease in Infants Less Than 90 Days: Case Series
Source: Front Pediatr. 2021 Jul 12;9:674899. doi: 10.3389/fped.2021.674899 (PMC8311174; doi:10.3389/fped.2021.674899)
Supplement: Supplementary file 1 [file Table_3.DOCX]

| Supplementary Table: Comparison of infants’ profiles based on their age | | | |
| --- | --- | --- | --- |
|  | Neonatal | Post neonatal | P |
| N | 16 | 20 |  |
| Age at presentation, days; median (range) | 17.5 (1–27) | 61 (29–90) | NA |
| Inborn | 6 | 15 | 0.013 |
| Males | 11 | 9 | NS |
| SVD | 10 | 16 | NS |
| BW, g | 2,980 (577-3,800) | 3,028 (2,530-3,680) | NS |
| Gestational age, weeks; median (range) | 38 (23-41) | 38 (37-40) | NS |
| Apgar’s scores at 5^th^ min; median (range) | 9 (4-9) | 9 (9-10) | NS |
| Mother’s COVID-19 status positive at birth | 1 | 1 | NS |
| Mother’s COVID-19 status positive at presentation | 5 | 11 | NS |
| Mothers exposed to COVID19 | 4 | 10 | NS |
| Infants exposed to COVID 19 | 10 | 12 | NS |
| Any breast feeding | 5 | 13 | 0.023 |
| Presentation | | | |
| Asymptomatic | 1 | 1 | NS |
| Fever | 9 | 16 | NS |
| Respiratory signs | 11 | 11 | NS |
| Gastrointestinal signs | 6 | 5 | NS |
| Lethargy/hypoactivity | 1 | 2 | NS |
| Hypoxia | 1 | 1 | NS |
| Symptoms before presentation, days; median (range) | 2 (0-4) | 1 (0-20) | NS |
| Temperature, C | 37.7 (37-38.8) | 38 (35-39) | NS |
| Respiratory rate, bpm | 46 (30-66) | 42 (30-62) | NS |
| Heart rate, bpm | 156 (130-165) | 168 (137-210) | NS |
| Blood pressure, mmHg; mean (systolic/diastolic) | 53 (83/40) | 55 (85/45) | NS |
| O2 saturation, % | 98 (79-100) | 96 (38-99) | NS |
| Upper respiratory infection | 11 | 13 | NS |
| Lower respiratory infection | 3 | 1 | NS |
| Shock | 1 | 1 | NS |
| MIS-C | 0 | 2 | NA |
| Bacterial co-infection | 3 | 2 | NS |
| Other comorbidities (CHD) | 15 | 3 | <0.0001 |
| Laboratory and imaging | | | |
| Positive blood cultures | 0/15 | 0/16 | NA |
| Positive urine cultures | 2/15 | 0/16 | NS |
| Positive CSF cultures | 1/9 | 0/7 | NS |
| Abnormal CXR | 15 | 7 | <0.0001 |
| Patients’ Blood groups | | | |
| Rh positive | 10 | 18 | NS |
| A antigen positive | 2 | 10 | 0.02 |
| B antigen positive | 5 | 3 | NS |
| Management issues | | | |
| Hospitalized | 14 | 9 | 0.003 |
| Admitted to ICU | 3 | 1 | NA |
| Hospital stay, days; median (range) | 5.5 (2-75) | 2 (1-13) | NS |
| ICU stay, days; median (range) | 32.5 (18-47) | 44.5 (15-75) | NS |
| Antibiotics use, days, median (range) | 4 (2-13) | 2 (1-18) | NS |
| Patients needed Respiratory support and/or O2 therapy | 2 | 2 | NS |
| Respiratory support & O_2_ use, days; median (range) | 45 (15-75) | 75 (75-75) | NS |

NA, not applicable/analyzable; NS, not significant
